# Melatonin ameliorates diabetic hyperglycaemia-induced impairment of Leydig cell steroidogenic function through activation of SIRT1 pathway

**DOI:** 10.1186/s12958-022-00991-6

**Published:** 2022-08-12

**Authors:** Ping Wang, Shoubing Zhang, Shuai Lin, Zhengmei Lv

**Affiliations:** 1 Department of Histology and Embryology, School of Basic Medical Sciences, Anhui Medical University, Hefei, 230032 China; 2 Clinical Laboratory, Gongli Hospital of Shanghai Pudong New Area, Shanghai, China

**Keywords:** Diabetes mellitus, Steroidogenesis, Melatonin, SIRT1, Leydig cell

## Abstract

**Background:**

Diabetes mellitus (DM)-related complications are important health problems worldwide. The underlying mechanisms for diabetic male subfertility/infertility are considerably complicated and need to be unveiled for therapeutic intervention. Melatonin treatment was investigated to assess the beneficial effects on injured steroidogenic function in DM due to its regulatory roles in mitochondria and autophagy.

**Methods:**

Diabetic hyperglycaemia was induced in rats injected with streptozotocin (STZ, 55 mg/kg/d) or simulated in TM3 Leydig cell line cultured with medium containing 30 mM D-glucose. Then, diabetic rats or the TM3 cells under high glucose were treated with melatonin. The diabetic rats were randomly divided into diabetes mellitus group (DM group), insulin treatment group (DM + INS group) and melatonin treatment group (DM + MT group). The TM3 Leydig cells were divided into a normal glucose control group (NG group), a high glucose treatment group (HG group), and a melatonin treatment group (HG + MT group). Then, Sirt1 (silent mating type information regulation 2 homologue) 1 expression was knocked down by siRNA.

**Results:**

The results showed that hyperglycaemia induced a decline in steroidogenesis, accompanied by autophagy defects, mitochondrial dysfunction and oxidative stress, in rats in the DM group or TM3 Leydig cells in the HG group. Furthermore, reduced SIRT1 expression levels and hyperacetylation were found in Leydig cells of DM group. Melatonin treatment ameliorated hyperglycaemia-induced impairment of Leydig cell function with simultaneous stimulation of 5’-adenosine monophosphate activated protein kinase (AMPK)/SIRT1 activity and the expression of autophagy-related genes. With regards to mitochondrial function, it promoted mitochondrial biogenesis with elevated PGC-1α, NRF1 and mtTFA, improved mitochondrial morphology, increased BNIP3L-related mitophagy and alleviated oxidative stress. Further results revealed that knockdown of Sirt1 in Leydig cells prevented the protective effects provided by melatonin against high glucose treatment, and interestingly, neutralization of reactive oxygen species (ROS) by N-acetyl-L-cysteine pretreatment abolished the stimulatory effect of melatonin on AMPK/SIRT1 activity in Leydig cells and prevented the induction of autophagy and mitochondrial biogenesis in the context of high glucose, indicating that modulation of SIRT1 pathway by melatonin was closely linked to ROS levels and oxidative stress.

**Conclusions:**

These findings suggest that SIRT1 pathway plays essential roles in the pleiotropic actions of melatonin on Leydig cells and in the prevention of hyperglycaemia-induced steroidogenic dysfunction. The stimulatory action of melatonin on SIRT1 pathway is related to oxidative stress and its antioxidant property. Our data provide new evidence for the relationship of melatonin and SIRT1 pathway in the context of hyperglycaemia, and melatonin as a combination therapy may be useful to combat DM-related complications, especially male reproductive system injury.

## Introduction

Diabetes mellitus (DM) is one of the most common diseases characterized by hyperglycaemia, and many people suffer from DM-related complications, including male infertility and sexual dysfunction [1]. The underlying pathological mechanisms of diabetic male reproductive injury include dysregulation of the hypothalamic-pituitary–gonadal (HPG) axis, increased DNA damage, oxidative stress, increased endoplasmic reticulum stress and impaired mitochondrial function [1, 2]. Multiple alterations in the reproductive function of male diabetic patients have been observed, including a decline in sperm parameters and testosterone levels [3]. At present, due to an increasing number of adolescents and men of reproductive age affected by DM [4], intensive investigation of the molecular mechanisms and therapy for diabetic male reproductive injury is warranted.

Testosterone is synthesized mainly in Leydig cells, which have rather high autophagic activity [5]. Autophagy is involved in testosterone synthesis by promoting the uptake of cholesterol, the raw material to produce testosterone [6]. However, under hyperglycaemic conditions, increased glucose oxidation overburdens mitochondria, resulting in electron leakage and increased reactive oxygen species (ROS) production, which irreversibly oxidizes DNA and cellular biomolecules and is detrimental to spermatogenesis [1, 7]. ROS can act as inducers of autophagy to remove damaged biomolecules and even organelles, such as dysfunctional mitochondria [8]. Because mitochondria are important sites for testosterone synthesis, mitochondrial dysfunction will affect testosterone synthesis [9]. Therefore, mitochondria are ideal targets for drug development to ameliorate DM-induced male reproductive function impairment, particularly the decline in testosterone levels.

Melatonin (N-acetyl-5-methoxytryptamine), the main hormone secreted by the pineal gland involved in energy balance maintenance, mitochondrial homeostasis and many other physiological processes [10], is also secreted by reproductive organs, such as the ovary and testis [11], indicating its potential role in reproductive activities. Melatonin is believed to be involved in male reproduction by modulating steroid hormone secretion and spermatogenic cell proliferation and protecting the testis against hyperthermia, environmental toxins, and drug-induced damage [11]. In the testes of diabetic mice, melatonin can alleviate Leydig cell apoptosis [12]. Moreover, melatonin, as an antioxidant, is targeted to mitochondria [10] and preserves mitochondrial function in type 1 diabetic rats with myocardial ischaemia/reperfusion injury [13], and by activating SIRT1/PGC-1alpha pathway, it even improves mitochondrial function in cadmium-induced hepatotoxicity [14]. Silent information regulator 1 (SIRT1), a nicotinamide adenine dinucleotide (NAD^+^)-dependent deacetylase, plays a key role in many metabolic and physiological processes, such as insulin resistance, oxidative stress, ageing and energy balance [15]. SIRT1 is a positive regulator of autophagy [16], and it promotes mitochondrial biogenesis through deacetylation and activation of PGC-1a, a master regulator of mitochondrial biogenesis that coactivates the nuclear respiratory factors (NRF1 and NRF2), which induce the transcription of genes involved in mitochondrial biogenesis [17].

Exogenous melatonin treatment in cell lines, rodent models, and diabetic patients has shown a potent effect in alleviating DM-related complications, such as diabetic cardiovascular disorders, neuropathy, retinopathy, and nephropathy [18]. In addition, melatonin is closely associated with male reproductive function, especially in the testis, because melatonin receptor is expressed in testicular cells and Leydig cells are sensitive to melatonin [19]. It is not yet well established from the existing data whether melatonin treatment is beneficial for reproductive function in male DM patients and how it can act to rehabilitate impaired steroidogenesis [20, 21]. Here, we demonstrate that melatonin mitigates hyperglycaemia-induced impairment of the steroidogenic function of Leydig cells both in vivo and in vitro with simultaneous activation of SIRT1 pathway. SIRT1 is required for the protective effect of melatonin on steroidogenesis in high glucose conditions, and the stimulation of AMPK/SIRT1 activity by melatonin is dependent on ROS levels. Our data provide new evidence for the relationship between melatonin and SIRT1 pathway in the context of hyperglycaemia, which functions to promote steroidogenesis by coordinating mitochondrial biogenesis, mitophagy and redox signalling.

## Materials and methods

### Reagents

Melatonin was obtained from Selleckchem (USA). D-Glucose was obtained from Sigma-Aldrich (St. Louis, MO, USA). NAC (N-acetyl-L-cysteine) was purchased from Beyotime Biotechnology (Shanghai, China). The primary antibodies against STAR, P450, AMPK, P-AMPK, COXIV, Cytc, NRF1, Beclin-1, ATG7, ATG12, ATG5 and LC3 were obtained from Cell Signaling Technology (Beverly, MA, USA); 3β-HSD, SIRT1, mtTFA, ATPB, PGC1-α and acetyl-Lysine were purchased from Abcam Biotechnology (Cambridge, MA, USA); BNIP3L, SOD2 and Tubulin 1-α were obtained from Beyotime Biotechnology (Shanghai, China); and LXR, GPX4, GPX5 and Actin were purchased from Proteintech Group (Wuhan, China). The goat anti-rabbit and goat anti-mouse secondary antibodies were purchased from Zhongshan Gold Bridge Biotechnology Co. Ltd. (Beijing, China). Alexa Fluor 488 donkey anti-mouse Immunoglobulin G (IgG) (H + L), MitoTracker™ Green FM and MitoSOX™ Red Mitochondrial Superoxide Indicator were obtained from Invitrogen (Carlsbad, CA, USA). The goat anti-mouse IgG/Alexa Fluor/594 were purchased from Zhongshan Gold Bridge Biotechnology Co. Ltd. (Beijing, China). Membrane and Cytosol Protein Extraction Kit, Cell Mitochondria Isolation Kit, Reactive Oxygen Species Assay Kit and Mitochondrial membrane potential assay kit with JC-1 were obtained from Beyotime Biotechnology (Shanghai, China).

### Establishment of type 1 diabetic model

Type 1 diabetic model was established as described previously [13]. Briefly, two-month-old Sprague–Dawley (SD) rats were intraperitoneally injected with streptozotocin (STZ, 55 mg/kg/d). Rats with fasting blood glucose greater than or equal to 16.7 mmol/L were identified as diabetic. These diabetic rats were randomly divided into three groups. One group was set as diabetes mellitus group (DM group) without any additional treatment. The second and third groups of diabetic rats that were treated respectively with insulin (subcutaneous injection, 1 unit/day) and melatonin (intraperitoneal injection, 10 mg/kg/d) for sixteen weeks were set as insulin treatment group (DM + INS group) and melatonin treatment group (DM + MT group), using the DM + INS group as positive control. Another group of normal rats without any treatment was set as control group (Con group). Then, body weight and fasting blood glucose were measured once a week for 21 weeks. All experimental and surgical procedures were approved by the Experimental Animal Ethics Committee of Anhui Medical University (No. LLSC20200006).

### Histological analysis

Rat testis tissue was collected and fixed in 4% paraformaldehyde solution. The samples were dehydrated in ethanol at different concentrations. After clearing with dimethylbenzene, the samples were embedded in paraffin blocks. The blocks were cut into continuous sections with 5 μm thickness for future use. For immunohistochemical staining, after the sections were dewaxed and rehydrated, they were heated in a microwave in citrate buffer solution for antigen retrieval. After resuming to room temperature, the sections were rinsed with PBS and incubated overnight with the primary antibody at 4 °C. The next day, the samples were incubated with secondary antibody for 1 h at room temperature, washed with PBS, stained with 3,3’-diaminobenzidine (DAB), restained with haematoxylin, sealed, and observed under a light microscope (Nikon, Eclipse 80i, Japan) and photographed.

### Electron microscopy

The testicular tissues of SD rats in each group were placed immediately into 2.5% glutaraldehyde fixation solution at 4 °C according to routine steps, then fixed with 1% osmic acid after PBS washing, embedded, and ultrathin sections were prepared. After double staining with uranyl acetate and lead citrate, the ultrastructural changes in testicular tissues in each group were observed in Hitachi HT7700 electron microscope and photographed.

### Cell culture

TM3 mouse Leydig cell line purchased from American Type Culture Collection (ATCC) Cell Bank of the Chinese Academy of Sciences was cultured in Dulbecco’s modified Eagle’s medium (DMEM)/F12 medium supplemented with 10% foetal bovine serum (FBS). TM3 cells of normal glucose control group (NG group) and high glucose treatment group (HG group) were treated respectively with 5.5 mM and 30 mM D-glucose (Sigma, USA) for 48 h; an HG group treated with melatonin (SelleckChem, USA) for 6 h at a concentration of 100 μM was set as the high glucose and melatonin treatment group (HG + MT group). An HG + MT group of TM3 cells whose Sirt1 expression was knocked down by siRNA was set as HG + MT + siSirt1 group (siSirt1 group). Another HG + MT group of TM3 cells was treated with ROS inhibitor N-acetyl-L-cysteine (NAC) for 48 h at a concentration of 5 μM with 30 mM D-glucose (HG + MT + NAC group).

### Western blot analysis

Total proteins were extracted with radioimmunoprecipitation assay (RIPA) lysis buffer containing 1 mM phenylmethyl sulfonyl fluoride (PMSF), and the protein concentration was measured with a bicinchoninic acid (BCA) protein assay kit. Nucleoprotein extraction kits (Beyotime, Shanghai, China) were used to extract nuclear proteins, and mitochondrial proteins were also extracted using a mitochondrial extraction kit (Beyotime, Shanghai, China). Proteins were separated by 6–15% sodium dodecyl sulfate–polyacrylamide gel electrophoresis (SDS-PAGE) and then transferred to PVDF membranes. After blocking with 10% milk for one to two hours, the membrane and primary antibody were incubated overnight at 4 °C. After washing with tris-buffered saline with Tween 20 (TBST), the membrane and secondary antibody were incubated together at room temperature for one to two hours. Protein bands were imaged using electrochemiluminescence (ECL) luminescent solution.

### Reactive oxygen species (ROS) detection

The levels of intracellular ROS production were observed by the fluorescent dye dichlorodihydrofluorescein dihydrate (DCFH-DA). Cells were inoculated in a 6-well plate at an appropriate density, treated and incubated in DMEM/F12 serum-free medium containing 10 μM DCFH-DA for 30 min in the dark at 37 °C. After washing with PBS, a positive fluorescence microscope (Nikon, Eclipse 80i, Japan) was used to observe and take pictures.

### Mitochondrial membrane potential measurement

A mitochondrial membrane potential assay kit with JC-1 fluorescent probe was used to detect changes in the cellular mitochondrial membrane potential in TM3 cells. When the cell fusion rate reached 70–80%, 10 μM of JC-1 staining solution was added to TM3 cell culture dishes in different treatment groups and incubated at 37 °C for 20 min with protection from light. Then the cells were washed twice with JC-1 staining buffer (1X), and following pre-warmed PBS buffer. The results were observed and photographed under a laser confocal microscope (Leica, TCS SP5, Germany).

### Evaluation of mitochondrial morphology

Cells were cultured in a dish at an appropriate density and incubated in DMEM/F12 serum-free culture medium containing 100 nM MitoTracker™ Green FM (Invitrogen, USA) for 30 min in the dark at 37 °C. The dye solution was replaced with fresh preheated culture medium, and the cells were observed and photographed under a laser confocal microscope (Leica, TCS SP5, Germany) to analyse the morphology of mitochondria in TM3 cells under different treatment conditions.

### Detection of mitochondrial ROS

Cells in a 6-well plate at an appropriate density were sequentially incubated in Hanks’ balanced salt solution (HBSS) containing 5 µM MitoSOX™ Red mitochondrial superoxide indicator (Invitrogen, USA) for 10 min at 37 °C, washed gently with PBS three times, preserved in preheated HBSS, observed and photographed under a positive fluorescence microscope (Nikon, Eclipse 80i, Japan).

### Immunofluorescence staining

Cells were cultured on slides, treated, fixed in 4% paraformaldehyde solution for 15 min, permeated with 0.2% Triton X-100 for 10 min, sealed at room temperature for 1 h with 2% bovine serum albumin (BSA), and then incubated overnight with primary antibody at 4 °C. After secondary antibody incubation for one to two hours in the dark at room temperature, nuclei were dyed with diamidino-2-phenylindole (DAPI) for 10 min. The slides were sealed with anti-quenching agent and observed under a positive fluorescence microscope (Nikon, Eclipse 80i, Japan) or laser confocal microscope (Leica, TCS SP5, Germany). The fluorescence intensity was analysed using Image-Pro Plus 6.0 software (Media Cybernetics).

### MDC staining

Cells were seeded into a 6-well plate at an appropriate density and incubated in PBS containing 3.5 mM monodansylcadaverine (MDC) (Sigma, USA) for 20 min in the dark at 37 °C. After washing gently in PBS three times, the cells were observed under a laser confocal microscope (Leica, TCS SP5, Germany) and photographed.

### SiRNA knockdown

Sirt1 siRNA was used to knock down Sirt1 in TM3 cells. Specific Sirt1 siRNAs and mismatched oligonucleotides in the control group were purchased from GenePharma (Shanghai, China). The Sirt1 siRNA sequence was GCACUAAUUCCAAGUUCUATT (sense) and UAGAACUUGGAAUUAGUGCTT (antisense). The experiment was carried out according to the instructions. In short, the liposomes Lipo3000 and Opti-MEM were mixed, and then the specific siRNA was mixed with Opti-MEM, adjusting the concentration of siRNA to 1 µM. Finally, these two mixtures were also mixed. After standing for 20 min, the mixture was added to the culture dish and cultured for 72 h. Cellular proteins were extracted for Western blot detection, or the cells were used for immunofluorescence staining.

### Statistical analysis

Grey values for Western blot and the average fluorescence intensity of the pictures were measured by ImageJ software. All data are presented as the mean ± standard error (SE). All measured data were subjected to one-way analysis of variance (ANOVA) followed by Bonferroni post hoc test with GraphPad Prism software version 6.07. *P* < 0.05 was considered statistically significant.

## Results

### Diabetic hyperglycaemia-induced decline in testosterone production and the beneficial effects of melatonin treatment

**Fig. 1:**
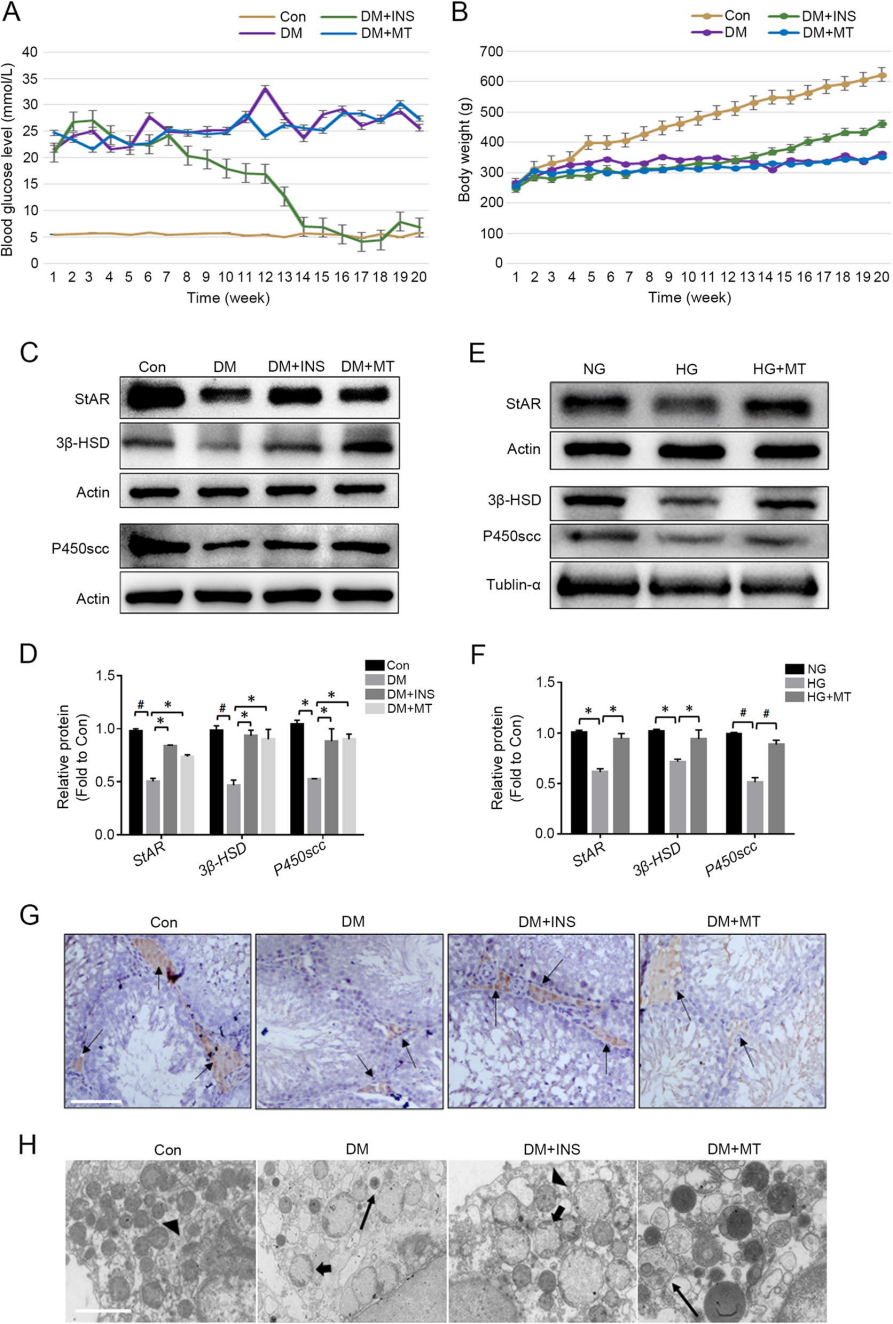
Melatonin alleviated diabetic hyperglycemia-induced impairment of testosterone synthesis in Leydig cells. **A** Changes in blood glucose level of SD rats. **B** Body weight changes of SD rats. **C**-**D** Protein expression level detection of StAR, 3β-HSD and P450scc in testicular tissues of rats in each group by Western blot and the statistical graph of gray value analysis; **E**–**F** Protein expression level detection of 3β-HSD and P450 in TM3 cells by Western blot and gray value analysis. **G** Immunohistochemical staining of rat testis. Arrow indicates Leydig cells labelled by 3β-HSD antibody. **H** Ultrastructure of Leydig cells in rat testis. Triangle indicates normal mitochondria, long arrow indicates autophagosomes, and short arrow indicates damaged mitochondria. Con, control group; DM, diabetes mellitus group; DM + INS, insulin treatment group of diabetic rats; DM + MT, melatonin treatment group of diabetic rats; NG, normal glucose control group; HG, high glucose treatment group; HG + MT, high glucose and melatonin treatment group; **D**-**F**, Data are expressed as fold change relative to Con. *, *P* < 0.05; #, *P* < 0.01. Bar: **G**, 50 μm; **H**, 2 μm

Type 1 diabetes mellitus model was successfully established in rats. Based on our previously published work [22], changes in blood glucose levels and body weight after melatonin treatment were examined. Unlike the insulin treatment group, the blood glucose level (Fig. 1A) and body weight (Fig. 1B) of rats in the DM + MT group did not show obvious changes compared with the DM group, indicating that melatonin treatment did not affect the blood glucose level and body weight of the rats. To test the effect of diabetic hyperglycaemia on testosterone production and whether melatonin helps improve testosterone production, the expression levels of steroidogenic acute regulatory protein (StAR), 3β-hydroxysteroid dehydrogenase (3β-HSD) and P450scc in rat testicular tissue were detected (Fig. 1C and D). Western blot results revealed that the expression of all three proteins decreased significantly in the DM group, but both melatonin and insulin treatment reversed their expression. In addition, Western blot results further showed that in TM3 cells, the expression levels of StAR, 3β-HSD and P450scc in the HG + MT group increased in comparison with the HG group (Fig. 1E and F). Immunohistochemical staining of rat testis tissue also suggested that 3β-HSD expression in the DM + MT group increased in comparison with the DM group (Fig. 1G). Electron micrography showed that mitochondria degenerated obviously in the DM group and was significantly recovered in the DM + INS and DM + MT groups (Fig. 1H). These data demonstrate that although melatonin did not decrease the blood glucose level, it ameliorated the high glucose-induced impairment of Leydig cell steroidogenesis.

### Melatonin prevented mitochondrial dysfunction induced by hyperglycaemia via simultaneous stimulation of AMPK/SIRT1 activity

**Fig. 2:**
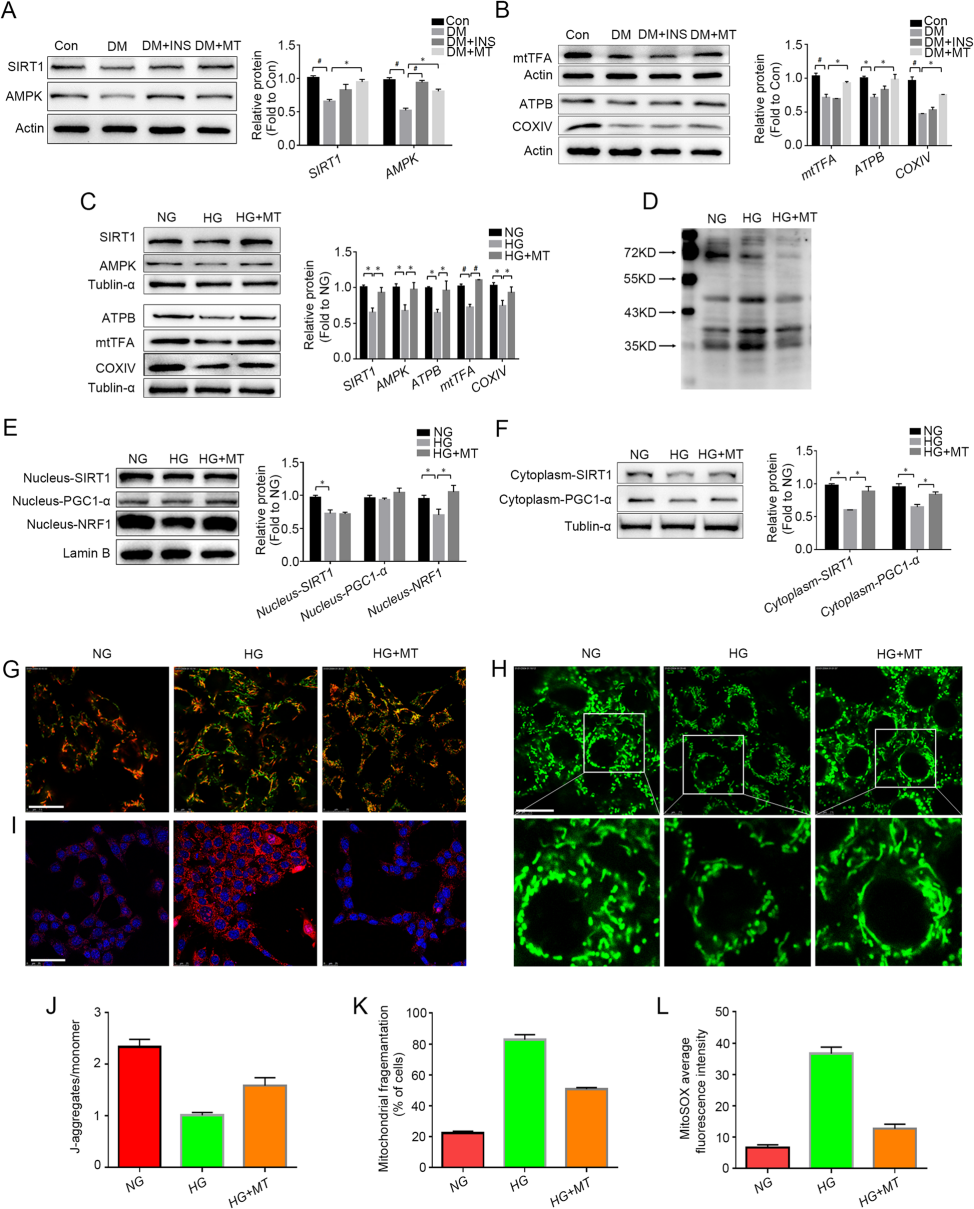
Effects of melatonin on AMPK/SIRT1 pathway dysregulation and mitochondrial dysfunction induced by high glucose. **A** The expression levels of SIRT1 and AMPK in rat testicular tissue detected by Western blot and the statistical graph of gray value analysis. **B** The expression levels of mitochondrial function-related proteins (mtTFA, ATPB and COXIV) in rat testicular tissue were detected by Western blot and their gray values were analyzed. **C** The expression levels of SIRT1, AMPK and mitochondrial function-related proteins in TM3 cells detected by Western blot and the statistical graphs of gray value analysis. **D** Acetylation level of total proteins in TM3 cells of each group was detected. **E**–**F** Protein levels of SIRT1, PGC-1α and NRF1 in cytoplasm and nucleus of TM3 cells were detected by Western blot and gray values of the protein bands were analyzed. **G** and **J** Mitochondrial membrane potential detection of TM3 cells by JC-1 probe and the ratio of J-aggregates to monomer (green fluorescence) was statistically analyzed. **H** and **K** Detection of the number and morphology of mitochondria in TM3 cells by MitoTracker and the percentage of cells with mitochondrial fragmentation (*n* = 150) was statistically analyzed. **I** and **L** Detection of mitochondrial reactive oxygen species in TM3 cells by MitoSOX and average fluorescence intensity analysis. Con, control group; DM, diabetes mellitus group; DM + INS, insulin treatment group of diabetic rats; DM + MT, melatonin treatment group of diabetic rats; NG, normal glucose control group; HG, high glucose treatment group; HG + MT, high glucose and melatonin treatment group; **A-B**, Data are expressed as fold change relative to Con; **C**, **E**–**F**, Data are expressed as fold change relative to NG. *, *P* < 0.05; #, *P* < 0.01. Bar: **G**-**H**, 15 μm; **I**, 50 μm

To explore the mechanism by which melatonin protects steroidogenic function against hyperglycaemia, the expression of SIRT1 and AMPK in rat testes was detected. Western blot results showed that the protein levels of both SIRT1 and AMPK decreased in the DM group, but they were upregulated in the DM + MT group and DM + INS group (Fig. 2A). Because mitochondrion is the main site of testosterone synthesis, we further detected the expression of mitochondrial transcription factor A (mtTFA), ATP synthase subunit beta (ATPB), cytochrome c oxidase subunit IV (COXIV) and cytochrome c (Cytc), important proteins that fulfil normal mitochondrial function, in rat testes and found that the expression of mtTFA, ATPB and COXIV increased significantly in the DM + MT group compared with the DM group (Fig. 2B). These expression levels were in accordance with the results in Leydig cell line TM3 cells (Fig. 2C). Acetylation detection of total proteins in TM3 cells found that the acetylation level of the proteins in the HG group increased compared to the acetylation level of the proteins in the Con group, and the acetylation level in the HG + MT group was lower than the acetylation level in the HG group (Fig. 2D), which indicates that protein acetylation modification may be involved in the damage caused by high levels of glucose and the protective mechanism of melatonin. These results demonstrated that mitochondrial function was impaired in DM testes and TM3 cells under high glucose, but after melatonin treatment, SIRT1 pathway was activated, and mitochondrial function was considerably recovered.

Further Western blot results showed that compared with the HG group, although there was no increase in the protein levels of nuclear SIRT1 and its downstream peroxisome proliferator-activated receptor gamma coactivator 1alpha (PGC-1α) in the HG + MT group (Fig. 2E), cytoplasmic SIRT1 and PGC-1α increased (Fig. 2F). Notably, nuclear respiratory factor 1 (NRF1) protein levels in the nucleus increased significantly in the HG + MT group (Fig. 2E). Fluorescent JC-1 detection revealed that mitochondrial membrane potential (MMP) decreased in the HG group, and JC-1 aggregates were reformed in the HG + MT group, indicating the restoration of MMP after melatonin treatment (Fig. 2G and J). MitoTracker Green dyes, used as mitochondrial morphology probes, are sequestered by mitochondria with normal function. Evaluation by MitoTracker probes showed that there were more spheroid-shaped mitochondria with decreased fluorescence in the HG group, indicating more damaged and fragmented mitochondria under high glucose conditions (Fig. 2H and K). MitoSOX detection found that the production of mitochondrial reactive oxygen species (mtROS) in TM3 cells increased in the HG group but was reduced by melatonin treatment to almost normal levels (Fig. 2I and L), also indicating that melatonin prevented mitochondrial dysfunction caused by high levels of glucose. These results demonstrated that melatonin activated SIRT1/PGC-1α/NRF1 signalling pathway, thus promoting the restoration of mitochondrial function.

### Melatonin stimulated autophagy and BNIP3L-related mitophagy under high glucose conditions

**Fig. 3:**
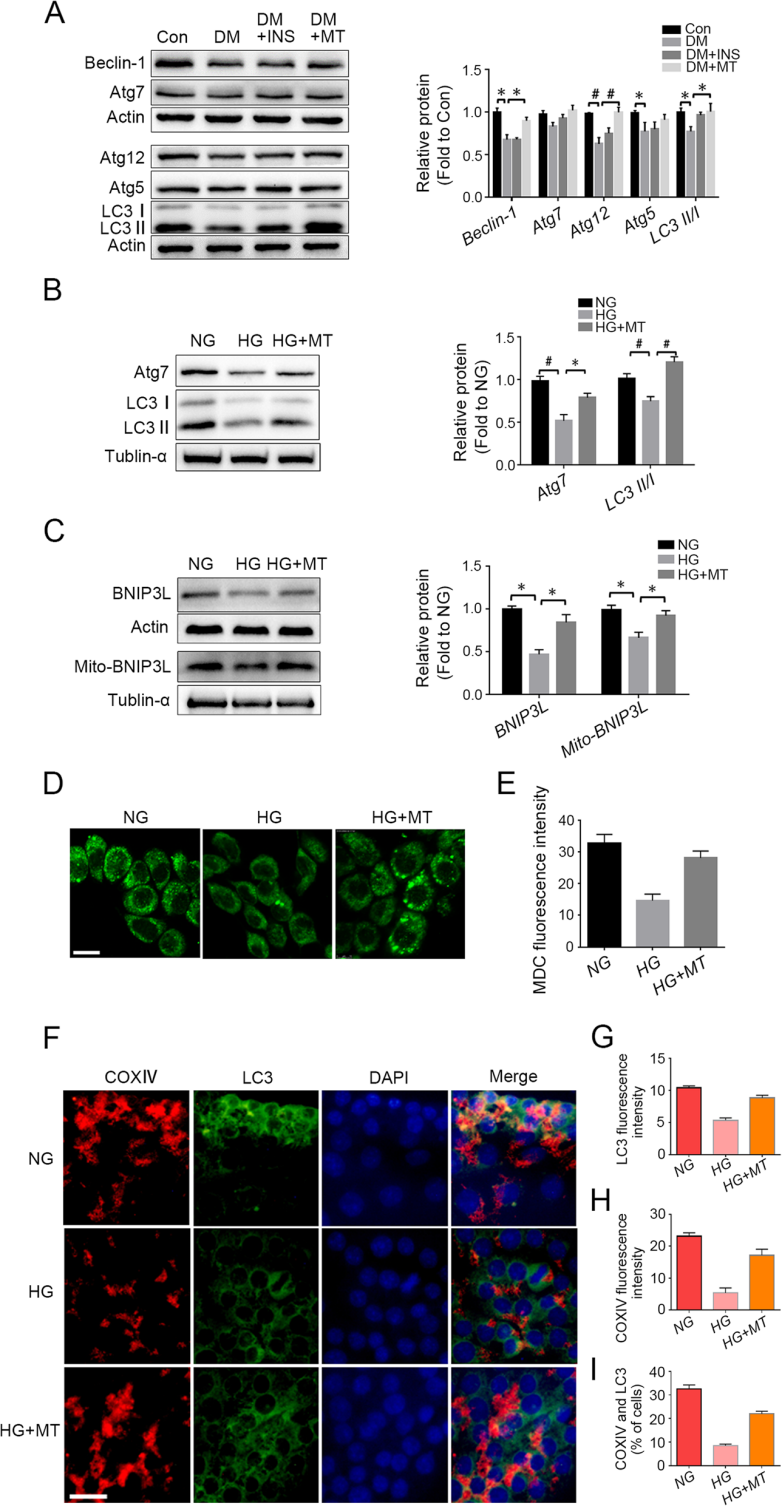
Melatonin prevented high glucose-induced decrease in autophagy and mitophagy. **A** Western blot was used to detect the expression levels of autophagy-related proteins in rat testicular tissue, and gray values were analyzed. **B** The expression levels of autophagy-related genes in TM3 cells detected by Western blot and statistical analysis of their gray values. **C** The expression levels of BNIP3L in total protein and mitochondrial protein of TM3 cells was detected by Western blot. **D**&**E** Autophagosome formation in TM3 cells was detected by MDC (monodansylcadaverine) staining and their fluorescence intensity was statistically analyzed. **F**-**I**. Immunofluorescent co-staining of COXIV and LC3 antibodies to detect mitophagy in TM3 cells and statistical analysis of mitophagy (randomly counting 150 cells). Con, control group; DM, diabetes mellitus group; DM + INS, insulin treatment group of diabetic rats; DM + MT, melatonin treatment group of diabetic rats; NG, normal glucose control group; HG, high glucose treatment group; HG + MT, high glucose and melatonin treatment group; **A**, Data are expressed as fold change relative to Con; **B**-**C**, Data are expressed as fold change relative to NG. *, *P* < 0.05; #, *P* < 0.01. Bar: **D**, 20 μm; **F**, 100 μm

Because autophagy mediates steroidogenesis regulation and Leydig cells show high autophagic activity [23], we detected the expression levels of autophagy-related proteins in rat testes (Fig. 3A) and TM3 cells (Fig. 3B). Western blot results revealed that the protein levels of Beclin-1, Atg12, and LC3 II decreased significantly in the testes of the DM group, and they were reversed to nearly normal levels after melatonin treatment (Fig. 3A). The results were similar for Atg7 and LC3 II/I in TM3 cells under high glucose conditions (Fig. 3B). Further detection by MDC also showed that autophagosome formation in TM3 cells decreased in the HG group, and this decrease was reversed by melatonin treatment (Fig. 3D and E). The mitophagy level in TM3 cells was assessed by Western blotting detection of the mitophagy receptor BCL2/adenovirus E1B interacting protein 3-like (BNIP3L/NIX) (Fig. 3C) and immunofluorescent colocalization of the mitochondrial marker COXIV and the autophagy marker LC3 (Fig. 3F-I). The results indicated that mitophagy activity decreased under high glucose conditions, but this decrease was reversed by melatonin treatment. Generally, these results demonstrated that hyperglycaemia decreased autophagy and mitophagy in rat testes of the DM group and TM3 cells, which is closely related to testosterone synthesis, and underlined the mechanism by which melatonin protects steroidogenesis against high levels of glucose.

### Sirt1 knockdown abolished the protective effect of melatonin against hyperglycaemia-induced collapse of steroidogenesis

**Fig. 4:**
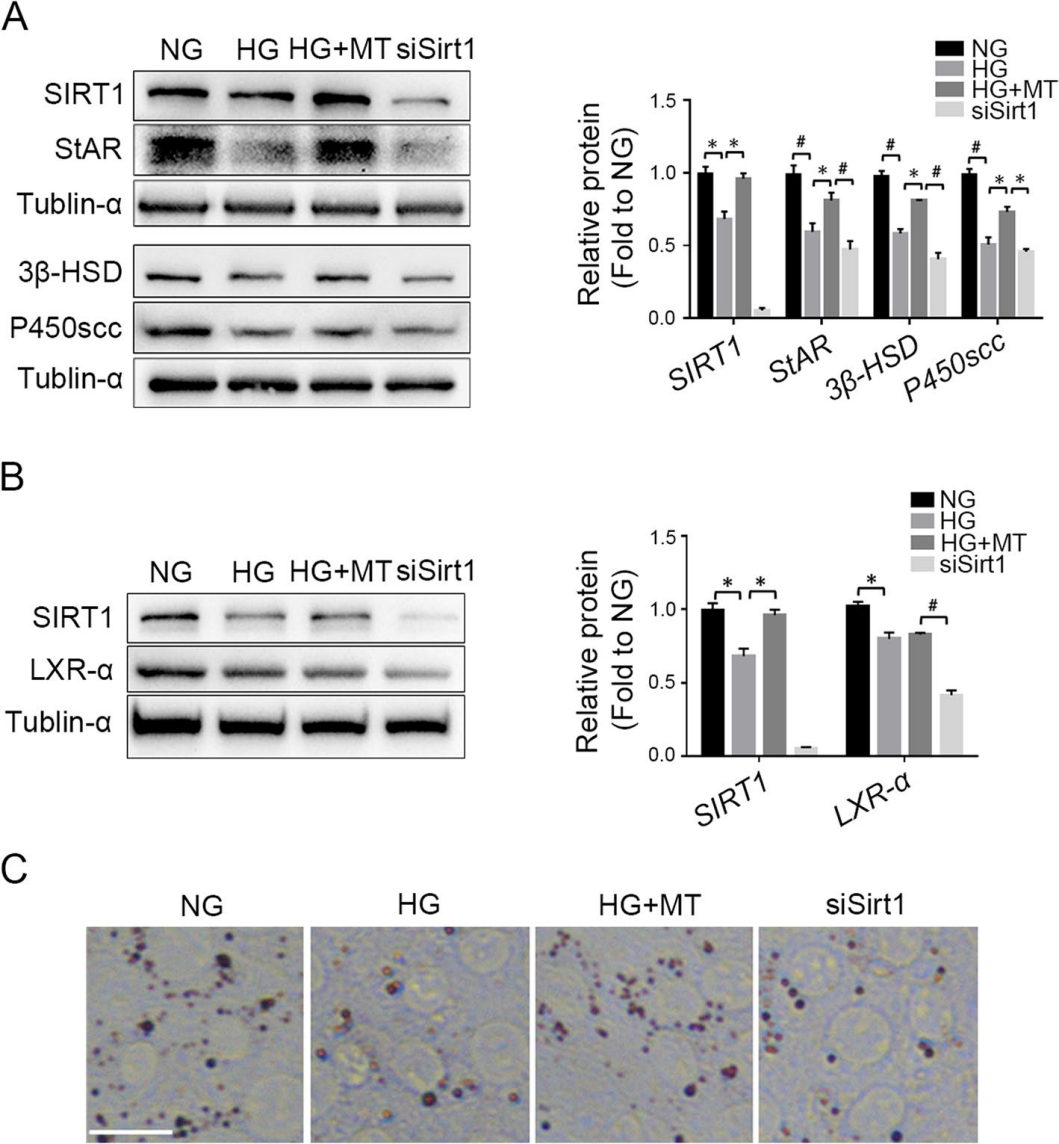
Sirt1 knockdown abolished melatonin protection of steroidogenesis against high glucose damages. **A** The protein levels of SIRT1, StAR, 3β-HSD and P450scc in TM3 cells were detected by Western blot and gray values were analyzed. **B** Western blot detection of SIRT1 and LXR-α expression in TM3 cells and gray value analysis of the protein bands. **C** Oil red staining was used to detect lipid droplets in TM3 cells. NG, normal glucose control group; HG, high glucose treatment group; HG + MT, high glucose and melatonin treatment group; siSirt1, HG + MT + siSirt1 group. **A**-**B**, Data are expressed as fold change relative to NG. *, *P* < 0.05; #, *P* < 0.01. Bar: **C**, 50 μm

To further confirm the role of SIRT1 pathway in the protective effect of melatonin treatment on steroidogenesis impaired by high glucose, we knocked down the expression of Sirt1 in TM3 cells, followed by HG and melatonin treatment. Western blot results showed that after Sirt1 knockdown, the protein levels of StAR, 3β-HSD and P450scc decreased significantly compared with the protein levels in the HG + MT group (Fig. 4A), indicating a decline in steroidogenesis induced by Sirt1 knockdown. In addition, the protein level of liver X receptor α (LXR-α), which activates genes involved in cholesterol transport and metabolism, was also downregulated after Sirt1 knockdown (Fig. 4B). In accordance with this result, Oil red O staining showed that lipid droplets were reduced in TM3 cells after Sirt1 knockdown in comparison with the HG + MT group (Fig. 4C). These results demonstrated that Sirt1 knockdown mitigated the protective effect of melatonin against hyperglycaemia-induced impairments in testosterone synthesis.

### Sirt1 knockdown weakens the protective effect of melatonin on mitochondrial function

**Fig. 5:**
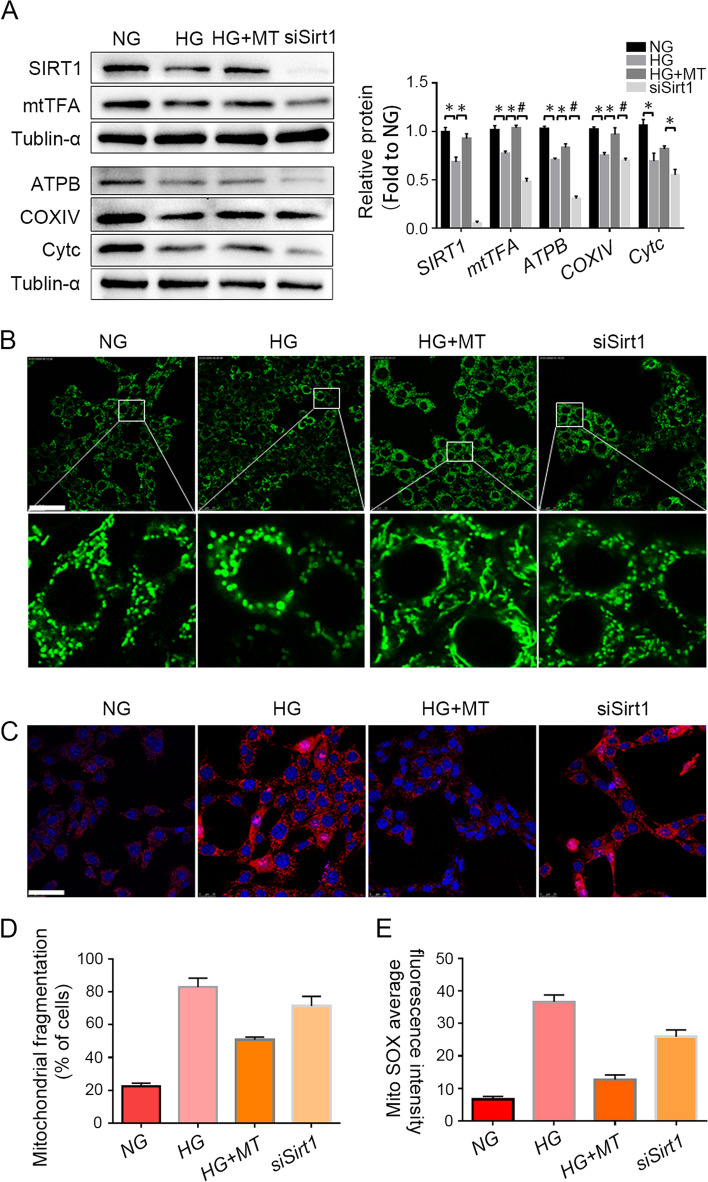
Sirt1 knockdown prevented the protective effect of melatonin on mitochondrial dysfunction caused by high glucose. **A** The expression levels of mitochondrial function-related proteins in TM3 cells were detected by Western blot and their gray values were analyzed. **B** and **D** Mitochondria in TM3 cells were stained by MitoTracker fluorescent probe and the percentage of cells with mitochondrial fragmentation (*n* = 150) was statistically analyzed. **C** and **E** Mitochondrial reactive oxygen species in TM3 cells were detected by MitoSOX, and the average fluorescence intensity was statistically analyzed. NG, normal glucose control group; HG, high glucose treatment group; HG + MT, high glucose and melatonin treatment group; siSirt1, HG + MT + siSirt1 group. **A**, Data are expressed as fold change relative to NG. *, *P* < 0.05; #, *P* < 0.01. Bar: **B**&**C**, 50 μm

To confirm that SIRT1 pathway is essential for melatonin to regulate mitochondrial function, the expression of Sirt1 in TM3 cells was knocked down, followed by HG and melatonin treatment. Western blot results showed that after Sirt1 knockdown, the levels of the mitochondrial functional proteins ATPB, mtTFA, COXIV and CytC significantly reduced, compared with those levels in the HG + MT group (Fig. 5A). Moreover, mitochondrial morphology was detected by MitoTracker probes (Fig. 5B and D), and mitochondrial ROS were assessed by MitoSOX (Fig. 5C and E). These results showed that elongated tubular-shaped mitochondria reduced significantly and mtROS levels increased in the Sirt1 knockdown group, compared with the HG + MT group. The results above demonstrated that SIRT1 pathway was essential for melatonin to ameliorate mitochondrial dysfunction caused by high glucose.

### Sirt1 knockdown blocks the regulation of melatonin on autophagy and mitophagy

**Fig. 6:**
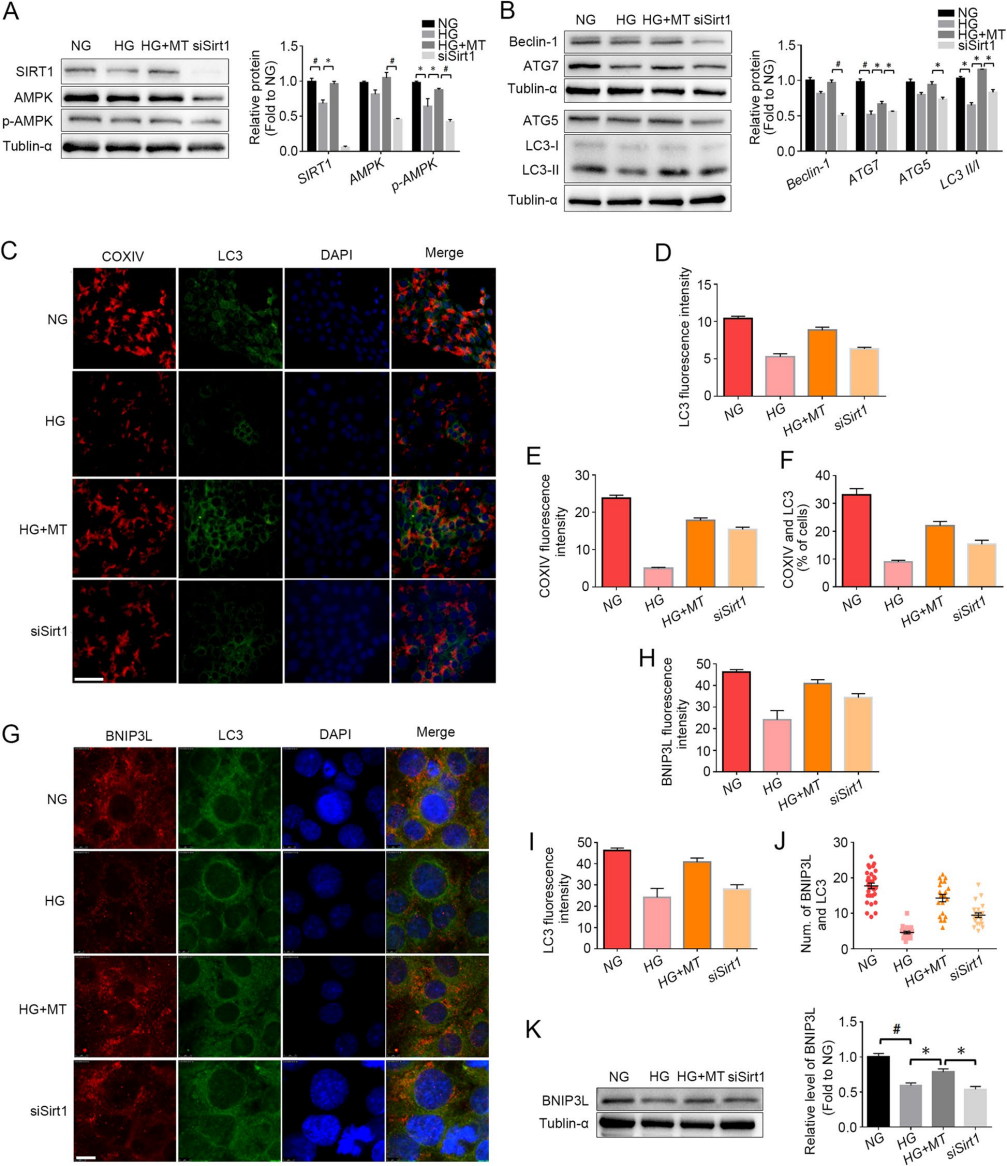
Sirt1 knockdown blocked the upregulation of melatonin on autophagy and mitophagy. **A** Expression levels of SIRT1 and AMPK in TM3 cells were detected by Western blot and their gray values were analyzed; **B** The expression levels of autophagy-related proteins Beclin-1, Atg7, Atg5 and LC3 in TM3 cells were detected by Western blot, and the gray values of protein bands were statistically analyzed; **C**-**F**. Mitophagy in TM3 cells was marked by COXIV and LC3 antibody co-staining. The fluorescence intensity and the percentage of co-stained cells (counting randomly 150 cells in six visual fields) were statistically analyzed. **G**-**J** Immunofluorescent co-staining of BNIP3L and LC3 antibodies in TM3 cells. The fluorescence intensity (randomly counting 100 cells in 10 visual fields) and the percentage of co-stained particles in the cells (totally 30 cells) were statistically analyzed. **K** The protein level of BNIP3L in TM3 cells was detected by Western blot, and the gray values of the protein bands were analyzed statistically. NG, normal glucose control group; HG, high glucose treatment group; HG + MT, high glucose and melatonin treatment group; siSirt1, HG + MT + siSirt1 group. **A**-**B**, Data are expressed as fold change relative to NG. *, *P* < 0.05; #, *P* < 0.01. Bar: **C**, 100 μm; G, 10 μm

To validate the role of SIRT1 in the regulation of autophagy and mitophagy after melatonin treatment, Sirt1 was knocked down by siRNA in TM3 cells, and then the cells were treated with HG and melatonin. After Sirt1 knockdown, the levels of AMPK and phosphorylated AMPK (p-AMPK) decreased (Fig. 6A), and autophagy-related proteins such as Beclin-1, Atg7, Agt5 and LC3 II were downregulated significantly in TM3 cells (Fig. 6B), compared with the HG + MT group. In addition, the immunofluorescence colocalization of the autophagy marker LC3 respectively with the mitochondrial marker COXIV (Fig. 6C-F) and the mitophagy marker BNIP3L (Fig. 6G-J) further demonstrated that mitophagy activity in TM3 cells was attenuated after Sirt1 knockdown. Moreover, downregulated BNIP3L was confirmed by Western blotting in TM3 cell, compared with the HG + MT group (Fig. 6K). These results demonstrated that melatonin regulated autophagy and mitophagy activity via SIRT1 pathway in the context of high glucose.

### Stimulation of SIRT1 pathway by melatonin is closely linked to ROS levels and oxidative stress

**Fig. 7:**
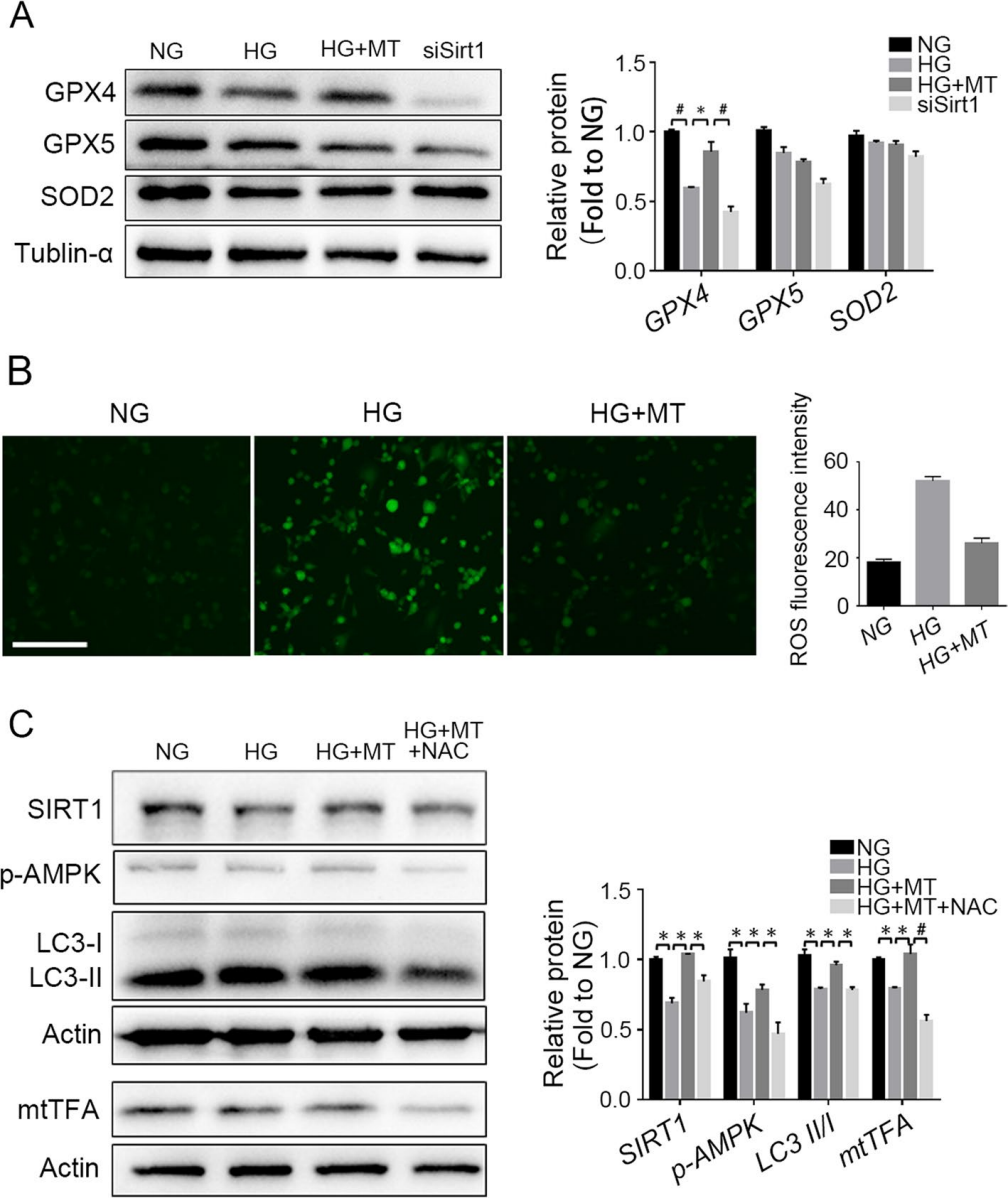
Stimulation of SIRT1 by melatonin was closely linked to ROS level and oxidative stress. In order to investigate the mechanisms by which melatonin stimulate SIRT1, ROS inhibitor NAC (N-Acetylcysteine) was used to pre-treat TM3 cells prior to melatonin treatment in high glucose condition. **A** The protein levels of antioxidant enzymes GPX4, GPX5 and SOD2 in TM3 cells detected by Western blot and statistical analysis of gray values. **B** ROS levels in TM3 cells were detected by DCFH-DA. The fluorescence intensity was analyzed statistically. **C** Expression levels of SIRT1, AMPK, autophagy marker LC3 as well as mitochondrial synthesis transcription factor mtTFA in TM3 cells of each group were detected by Western blot and gray values of protein bands were analyzed statistically. NG, normal glucose control group; HG, high glucose treatment group; HG + MT, high glucose and melatonin treatment group; siSirt1, HG + MT + siSirt1 group; HG + MT + NAC, HG + MT + NAC group. **A** and **C**, Data are expressed as fold change relative to NG. *, *P* < 0.05; #, *P* < 0.01. Bar: **B**, 100 μm

The antioxidant enzymes glutathione peroxidase 4 (GPX4), glutathione peroxidase 5 (GPX5) and superoxide dismutase 2 (SOD2) were detected by Western blotting, and ROS were assessed. Melatonin increased the antioxidant GPX4 level and reduced the ROS level in the HG + MT group compared with the HG group (Fig. 7A-B), and the ROS level in the HG group was much higher than the ROS level in the NG group (Fig. 7B), indicating that high glucose induced oxidative stress and that melatonin treatment effectively mitigated oxidative stress. SIRT1 knockdown significantly blocked the upregulation of GPX4 levels induced by melatonin (Fig. 7A). To determine whether the activation of SIRT1 pathway and autophagy by melatonin treatment is mediated by ROS signalling, the ROS inhibitor N-acetyl-L-cysteine (NAC) was used to pretreat cells under high glucose conditions prior to melatonin treatment. Melatonin prevented the decline in the protein levels of SIRT1, p-AMPK, mtTFA and LC3 II in the HG + MT group, compared with the HG group (Fig. 7C). Pretreatment with the ROS inhibitor NAC suppressed melatonin-induced upregulation of SIRT1, p-AMPK, mtTFA and LC3 II in the HG + MT + NAC group, compared with the HG + MT group (Fig. 7C), indicating that increased ROS mediated the regulation of melatonin on mitochondrial biogenesis and autophagy and, most interestingly, the stimulation of SIRT1. These results demonstrated that the antioxidant activities of melatonin are closely interconnected with SIRT1 upregulation and its actions on autophagy and mitochondrial function.

## Discussion

DM is a well-known risk factor for male infertility. Although the functions of melatonin in many physiological and pathological processes have been widely investigated, its therapeutic role in DM-related complications has not been completely explained. Here, we report that although melatonin does not change glycaemic levels, consistent with other report [24], it mitigates the impairment in steroidogenesis caused by hyperglycaemia in rat testes through activation of SIRT1 pathway, resulting in enhanced mitochondrial function, autophagy and mitophagy activity as well as mitigated oxidative stress. Our data revealed that in DM rat testes and mouse Leydig cell line TM3 cells under high glucose, testosterone production, SIRT1 expression, mitochondrial function and autophagic activity decreased significantly; however, depression of all these aspects was reversed by melatonin treatment, which was independent of its impact on blood glucose levels [24]. Furthermore, the protective effects of melatonin treatment were partially prevented after Sirt1 knockdown in TM3 cells, indicating that the pleiotropic actions of melatonin were closely dependent on SIRT1 pathway under high glucose conditions.

SIRT1, a member of the conserved NAD^+^-dependent deacetylase family, plays a key role in cellular metabolism. SIRT1 functions to regulate metabolic health by deacetylating many target proteins [25]. Our results found that the level of SIRT1 decreased in DM rat testes and TM3 cells under high glucose, consistent with a previous report [26], but melatonin treatment stimulated SIRT1 levels. Sirt1 knockdown partially abolished the protective effect of melatonin on high glucose-induced impairment of testosterone synthesis, further demonstrating that melatonin exerts its effect via SIRT1 pathway, and treatment with high glucose and melatonin together induced the simultaneous alteration of protein acetylation levels in TM3 cells. SIRT1 has been reported to contain two nuclear localization signals and two nuclear export signals, which enable it to shuttle between the nucleus and cytoplasm, and cytoplasmic SIRT1 inhibits cell migration and invasion in ovarian carcinoma [27]. In our study, Western blot analyses indicated both cytoplasmic and nuclear localization of SIRT1 in TM3 cells. Melatonin treatment stimulated cytosolic SIRT1 in a more significant way, although SIRT1 is considered predominantly located in the nucleus [28, 29]. SIRT1 has been reported to deacetylate mitochondrial SIRT3, which deacetylates and regulates many mitochondrial proteins [30], indicating that under high glucose conditions, cytoplasmic SIRT1 may have an important role in the protective effect of melatonin on steroidogenesis. Previous studies revealed that SIRT1 phosphorylation by AMPK leads to deacetylation and activation of PGC-1α, which coactivates NRF1 [31]. Other researchers reported that both AMPK phosphorylation and SIRT1 deacetylation can directly activate PGC-1α, which acts as a master regulator of mitochondrial biogenesis [32]. The expression of nuclear-encoded mitochondrial proteins is predominantly controlled by NRF1 and NRF2. Mitochondrial transcription factor A (mTFA) mediates mitochondrial DNA transcription and replication through regulation of NRF1/2 [17]. Our results revealed that melatonin treatment activated AMPK/SIRT1 pathway, with stimulation of NRF1, mtTFA and mitochondrial functional proteins such as ATPB, Cytc and COXIV. The fact that melatonin targets mitochondrial biogenesis via AMPK/SIRT1 pathway may explain many of the beneficial effects of melatonin on Leydig cell function in DM.

Energized and polarized mitochondria are required for testosterone synthesis in Leydig cells. The state of mitochondria may be involved in regulating steroidogenesis and is closely linked to the transfer of cholesterol into mitochondria, which is the rate-limiting step of testosterone synthesis [33]. The tight coordination of mitochondrial biogenesis and mitophagy functions to regulate mitochondrial turnover and preserve mitochondrial function [34, 35]. In Leydig cells, autophagy also mediates cholesterol trafficking, thus controlling steroid hormone production [6]. SIRT1 deacetylates the nuclear receptor LXR, which is a major regulator of whole-body cholesterol and lipid homeostasis [6, 36]. Our results showed that DM and melatonin treatment impacted LXR levels and lipid droplets in testicular tissues or TM3 cells. High glucose decreased autophagy in the testes of the DM group or TM3 cells, but this effect was reversed by melatonin treatment. The modulation of autophagy by melatonin is partially dependent on SIRT1. SIRT1 knockdown partially abolished the stimulatory effects of melatonin on the expression of autophagy-related proteins such as Beclin-1, Atg7, Agt5 and LC3 II. SIRT1 is an important positive regulator of autophagy and mediates the expression of many autophagy-related genes [37]. The mitophagy receptor BNIP3L, also named Nix, binds to LC3/GABARAP proteins, which function to remove damaged mitochondria [38]. The protein level of BNIP3L was also upregulated by melatonin treatment, consistent with the immunofluorescence analysis of mitophagy. Thus, melatonin modulates autophagy and mitophagy via SIRT1, thereby maintaining mitochondrial function, which is essential for mitochondrial steroidogenesis, and transportation of cholesterol, the raw material for steroid production.

Consistent with other reports [39], mitochondrial morphology was also improved by melatonin treatment in the context of high glucose. Mitochondria are highly dynamic organelles constantly undergoing two opposite processes of fusion and fission. Studies have proven that nutrient excess is a stimulus that leads to fragmented mitochondrial networks mediated mainly by fission, and fragmentation/fission is considered detrimental to mitochondrial function, whereas fused networks are thought to be beneficial, which seems to improve mitochondrial function and produce fewer ROS [35, 39]. Our work revealed that under high glucose conditions, mitochondrial dynamics transformed towards fragmentation, ROS levels increased, and mitophagy activity abated significantly. Excessive fission and depressed mitophagy dramatically mediated mitochondrial dysfunction, including reduced expression of mitochondrial function-associated proteins, decreased MMP and increased ROS levels, in accordance with a previous report [40]. We also found that treatment with melatonin improved mitophagy, facilitated mitochondrial fusion and decreased ROS production, and Sirt1 knockdown partially prevented these beneficial effects provided by melanin treatment in a high glucose context. Recently, melatonin has been shown to prevent Drp1-mediated mitochondrial fission through SIRT1-PGC1α pathway, which contributes to protection against diabetes-induced cardiac dysfunction [38]. In addition to mitochondrial dynamics, there are various mitochondrial quality control mechanisms interconnected and engaged in coordinating mitochondrial biogenesis and turnover, such as mitophagy [41]. Our results demonstrated that under high glucose conditions, melatonin and SIRT1 interact to regulate mitochondrial dynamics and maintain mitochondrial homeostasis, which benefits steroidogenesis in Leydig cells.

Melatonin provides protection against hyperglycaemia-induced impairments in steroidogenesis, with simultaneous stimulation of SIRT1 pathway. However, the underlying mechanisms by which melatonin upregulates SIRT1 are not yet clear. In our study, melatonin treatment reduced the excessive ROS and mtROS release found under high glucose conditions and increased antioxidant enzymes such as GPX4 to prevent oxidative stress. Sirt1 knockdown in TM3 cells partially abolished the antioxidant activities of melatonin and its regulatory roles in autophagy, mitophagy and mitochondrial biogenesis. Interestingly, pretreatment with the ROS inhibitor NAC, which neutralizes ROS, eliminated the upregulation of SIRT1, LC3 and mtTFA induced by melatonin treatment. These data revealed that SIRT1 modulation by melatonin may be related to its well-known antioxidant actions, probably through the oxidative stress pathway. Mitochondrial dysfunction results in an increase in ROS, which in turn activate autophagy to remove oxidative damage and mediate the pleiotropic actions of melatonin [7, 42]. It is clear now that the role of SIRT1 in regulating the redox environment is key for the cellular response to oxidative stress. Melatonin and SIRT1 seem to interact to regulate oxidative stress.

Nevertheless, our research has limitations. Since hyperglycaemia is the main manifestation and pathological factor of diabetes, in vitro culture medium supplemented with a high level of glucose is often used to study the molecular mechanism related to diabetes. However, the in vitro and in vivo regulation of glucose metabolism is different, leading to some difficulties in explaining the development of DM. The results obtained in experimental model animals may also be different from the mechanisms in the human body [43]. In addition, melatonin is a pleiotropic molecule regulating biorhythm and sleep, and its effects on body metabolism are diverse and complex. In this study, in vitro and in vivo research of melatonin focused on its beneficial effects on steroidogenesis by targeting diabetic manifestations such as mitochondrial dysfunction and oxidative stress, but many other questions, such as the appropriate dosage and timing of melatonin intake to optimize the effect of melatonin, still need to be considered in terms of its clinical application [18].

## Conclusions

In summary, SIRT1 pathway is required for melatonin to prevent hyperglycaemia-induced impairment of steroidogenesis in Leydig cells. Under high glucose conditions, melatonin upregulated the AMPK/SIRT1 activity to improve mitochondrial biogenesis, autophagy, BNIP3L-related mitophagy and mitochondrial dynamics. The protective effects of melatonin on mitochondrial function benefit steroidogenic machinery in Leydig cells in the context of hyperglycaemia. Stimulation of SIRT1 by melatonin in DM may explain the pleiotropic functions of melatonin in treating metabolic disorders. Moreover, modulation of SIRT1 by melatonin is dependent on ROS levels and closely linked to its antioxidant actions. Melatonin and SIRT1 may interact to coordinate mitochondrial biogenesis, mitophagy and redox signalling, which is beneficial for the steroidogenic function of Leydig cells in DM. Our data provide new evidence for the relationship of melatonin and SIRT1 pathway in the context of hyperglycaemia, and although melatonin did not decrease blood glucose levels as insulin did, it may be used as a supplement to combat diabetic complications such as diabetic male reproductive impairment.

## Data Availability

The data are available from the corresponding authors upon request.
